# Diamond-Blackfan anemia caused by chromosome 1p22 deletion encompassing *RPL5*

**DOI:** 10.1038/s41439-019-0067-5

**Published:** 2019-08-08

**Authors:** Makiko Tominaga, Satoshi Hamanoue, Hiroaki Goto, Toshiyuki Saito, Jun-ichi Nagai, Mitsuo Masuno, You Umeda, Kenji Kurosawa

**Affiliations:** 1 Division of Medical Genetics, Kanagawa Children’s Medical Center, Yokohama, Japan; 2 Children’s Medical Center, Showa University Northern Yokohama Hospital, Yokohama, Japan; 3 Division of Hematology and Oncology, Kanagawa Children’s Medical Center, Yokohama, Japan; 4 Department of Clinical Laboratory, Kanagawa Children’s Medical Center, Yokohama, Japan; 5 Genetic Counseling Program, Kawasaki University of Medical Welfare, Kurashiki, Japan

**Keywords:** Genetics research, Anaemia

## Abstract

Diamond-Blackfan anemia (DBA) is an inherited anemia with multiple congenital malformations, and mutations in ribosomal protein genes have been identified as the underlying cause. We describe a female patient with mild DBA due to 1p22 deletion, encompassing the gene encoding 60S ribosomal protein L5 (*RPL5*). Considering previously reported cases together with our patient, we suggest that *RPL5* haploinsufficiency might cause a less severe form of DBA than loss-of-function mutations.

Diamond-Blackfan anemia (DBA) [MIM. 105650] is an autosomal dominant disorder characterized by severe normochromic and macrocytic anemia with normal leukocytes and platelets, congenital malformations, and growth retardation. The phenotype varies from mild to severe fetal anemia^1^, and DBA is associated with an increased risk of hematological malignancy^1^. Mutations in 19 genes encoding ribosomal proteins have been recognized as causing DBA^2^. The mutations reported to date include single-nucleotide variants and copy-number variants, both of which result in loss-of-function or haploinsufficiency of the causal genes^2–6^. Although mutations in *RPL5*, encoding 60S ribosomal protein L5, account for 11% of the patients with DBA^2,7^, only three patients have been reported to have a large deletion of *RPL5*^4,6,8,9^. Here, we report a female patient with DBA caused by 1p22 deletion, and we attempt to elucidate the clinical and hematological features of this large deletion encompassing *RPL5*.

The proposita was a 20-year-old woman. She was born at 39-weeks gestation after an uneventful pregnancy. At birth, her weight was 2055 g (−2.3 SD), and her length was 48 cm (−0.2 SD); her occipitofrontal circumference (OFC) was 32 cm (−0.6 SD). She underwent ligation of the patent ductus arteriosus on day 27; total repair of her atrial septal defect occurred at 1 year 9 months. At her first visit to our genetics clinic at 3 years of age, her weight and height were 11.1 kg (−1.2 SD) and 84.4 cm (−2.3 SD), respectively, with an OFC of 52.4 cm ( + 2.5 SD). Her facial appearance characteristics included typical down-slanting palpebral fissures, deep-set eyes, a thin upper lip, and macrocephaly. Hypoplastic finger-like thumbs with nail hypoplasia were noted (Fig. 1a). Brain magnetic resonance imaging revealed cortical atrophy and dilated ventricles. Her developmental milestones were delayed, with head control at 9 months, rolling over at 1 year, and walking without support at 4 years. At the age of 6 years, she was noted as having mild anemia: hemoglobin (Hb), 10.5 g/dL; mean corpuscular volume (MCV), 89 fL; mean corpuscular hemoglobin (MCH), 30.2 pg; white blood cell (WBC) count, 5500/cumm; and platelet count, 32.2 × 10^4^. At the age of 16 years, her anemia continued, with values as follows: Hb, 8.8 g/dL; MCV, 92 fL; MCH, 31.8 pg; WBC count, 3200/cumm (lymphocytes 48%, neutrophils 41%); and platelet count, 32.4 × 10^4^. Her bone marrow had a markedly hypocellular appearance, with a small number of erythroid and myeloid cells and megakaryocytes. Normal values in cytogenetic stress testing were obtained for mitomycin C, bleomycin, cyclophosphamide, diepoxybutane, and fludarabine. She also had primary amenorrhea and underwent vaginal fenestration for hematometrocolpos due to vaginal atresia at 16 years. At age 20, the following were recorded:Hb, 9.5 g/dL; MCV, 92.7 fL; MCH, 30.4 pg; WBC count, 3100/cumm; and platelet count, 29.1 × 10^4^. To date, she has not required transfusion for her mild hypochromic anemia.

**Fig. 1:**
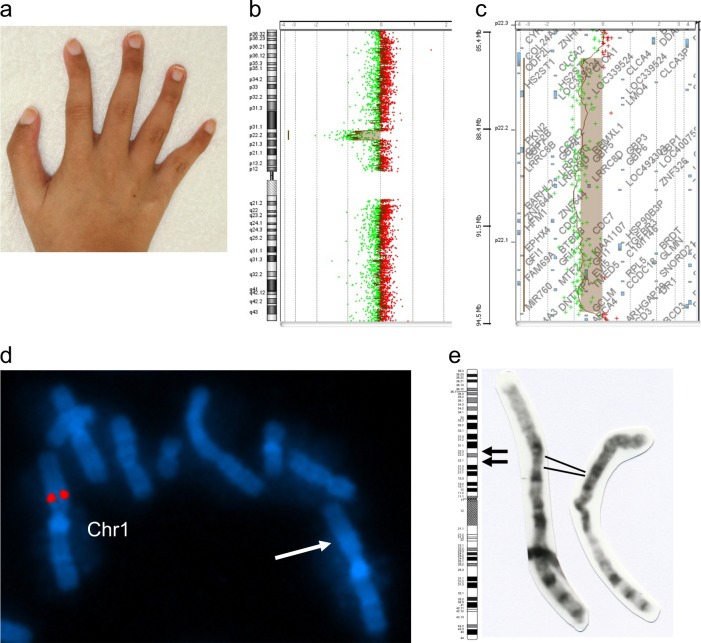
Clinical phenotype and molecular cytogenetic findings. **a** Hypoplastic thumb (triphalangeal thumb) was noted. **b**, **c** Array CGH analysis showing the 7.9-Mb deleted region at 1p22.1–p22.3. **d** Partial image of metaphase fluorescence in situ hybridization (FISH) of lymphocytes using the RP11–62M16 BAC clone (chr1: 92381303–92517650, NCBI35/hg17) as a specific probe for 1p22.1 (red). One signal was observed for the patient, consistent with a deletion at 1p22.1–p22.3. The signal of RP11–62M16 was absent from derivative chromosome 1 (arrow). **e** Retrospective evaluation of G-banded metaphase chromosome 1 revealed a heterozygous deletion of 1p22.1–p22.3 (arrows)

Written informed consent was obtained from the parents of the patient, and this study was performed in accordance with the Kanagawa Children’s Medical Center Review Board and Ethics Committee. Array comparative genomic hybridization (array CGH) using Agilent SurePrint G3 Human CGH Microarray Kit 8 × 60 K (Agilent Technologies, Inc., Santa Clara, CA, USA) revealed a 7.9-Mb deletion (arr[GRCh37] 1p22.3p22.1(86369841_94276387)x1) (Fig. 1b, c)^10^. No other genomic imbalances were identified based on the array analysis. Fluorescence in situ hybridization (FISH) analysis with relevant bacterial artificial chromosome (BAC) clones confirmed the deletion (Fig. 1d). Both parents refused cytogenetic evaluation. Further retrospective evaluation of G-banded metaphase chromosome 1 revealed a heterozygous deletion of 1p22.1–p22.3 (Fig. 1e).

The patient exhibited variable clinical manifestations, such as multiple congenital anomalies, moderate to severe developmental delay, and characteristic hematological findings of mild normochromic anemia and neutropenia. The clinical features overlapped with those of DBA and Fanconi syndrome. However, hematological analysis excluded the possibility of Fanconi anemia. The array CGH analysis revealed a 7.9-Mb deletion of 1p22.1–p22.3 encompassing 40 OMIM genes, including *RPL5*. To our knowledge, only three cases with large deletions of *RPL5* associated with DBA have been reported^4,6,8,9^. In general, most patients with DBA show a steroid-dependent or transfusion-dependent clinical course. Although the detailed clinical and hematological features of the three patients with *RPL5* haploinsufficiency are not available, two patients were reported to be steroid responsive^4,8^. Considering those cases together with our patient who showed a mild form of DBA without hematological treatments, we believe that *RPL5* haploinsufficiency might result in a less severe form of DBA than that caused by loss-of-function mutations. Although the involvement of neighboring genes could not be proven in the etiology of the patient’s phenotype, our case provides crucial information on the underlying mechanism for DBA (Fig. 2). Further information on DBA associated with genetic studies is required for a clearer understanding of the genetic and molecular bases of DBA.

**Fig. 2:**
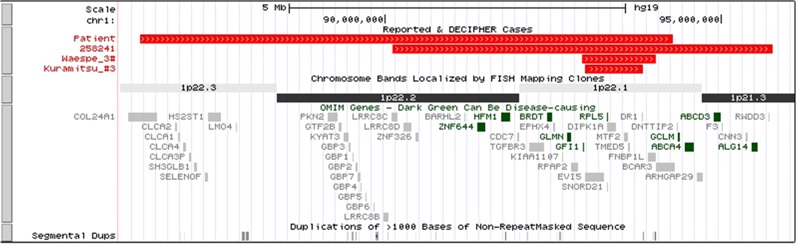
**Schematic representation of the 1p21.3–p22.3 deletions in the present case, DECIPHER patient (2258241), and previously reported cases encompassing** ***RPL5*** **based on USCS Genome Browser 2009 (GRCh37/hg19) Assembly** (http://www.genome.ucsc.edu)

## Data Availability

The relevant data from this Data Report are hosted at the Human Genome Variation Database at 10.6084/m9.figshare.hgv.2594.
